# Fluid Flow and Heat Transfer Performances of Aluminum Alloy Lattices with Triply Periodic Minimal Surfaces

**DOI:** 10.3390/ma18071407

**Published:** 2025-03-22

**Authors:** Zhensen Liu, Zetian Gao, Mingqiu Dai, Bingke Song, Biqi Yang, Tao Zhang, Shuangyin Yuan, Gang Liu, Miao Zhao

**Affiliations:** 1 Suzhou XDM 3D Printing Technology Co., Ltd., Suzhou 215000, China; 2 Shanghai Institute of Spacecraft Equipment, Shanghai 200240, China

**Keywords:** lattices, fluid flow characteristics, heat transfer characteristics, aluminum alloy

## Abstract

Thermal protection systems play a pivotal role in astronautical engineering fields. However, traditional rectangular fin (RF) structures exhibit low thermo-fluid properties. Inspired by the minimal surfaces in nature, this study develops three types of triply periodic minimal surface (TPMS) lattices, namely, sheet primitive (SP), network I-WP (NW), and sheet I-WP (SW) by using mathematical formulae. The TPMS lattices are fabricated by laser powder bed fusion using AlSi10Mg powder. A convective heat transfer simulation model of TPMS lattices is established and validated through experiments. The fluid flow characteristics, heat transfer characteristics, and overall heat transfer performance of the TPMS lattices are comprehensively investigated based on the simulation model. Results show that the relationship between pressure loss and flow velocity of the TPMS lattices satisfies the Darcy–Forchheimer law. Compared to traditional RF structures, the TPMS lattices exhibit a more uniform temperature distribution at the same flow rate, and the highest convective heat transfer coefficient is increased by approximately 96.62%. This is due to the complex internal structures of the TPMS lattices, which enhance the disturbance of the fluid flow and further improve the heat transfer coefficient. The overall thermal transfer index (α) of the TPMS lattices is higher than that of traditional RF structures with an order of $\alpha_{SP}>\alpha_{SW}>\alpha_{NW}>\alpha_{RF}$, which confirms the potential applications of TPMS lattices in thermal protection systems.

## 1. Introduction

The progressive development of space exploration technologies has driven spacecraft thermal management systems toward lightweight, high-strength, and thermally efficient configurations with increasing structural complexity and miniaturization requirements. Lattice architectures that synergistically combine structural integrity with multifunctional capabilities have emerged as promising candidates for advanced thermal regulation applications. The combination of lightweight [1], high-strength [2], and excellent thermal performances [3] positions lattices as a solution for next-generation spacecraft thermal management challenges.

Conventional lattices are composed of nodes and struts, where the arrangement of these elements can be modified to enhance mechanical and thermal properties [4,5,6]. For instance, Kaur et al. [7] investigated the heat transfer performance of conventional lattice structures in air and found that they can weaken thermal boundary layers and significantly enhance heat transfer. Yan et al. [8] designed X-shaped lattices that induce substantial secondary flows, thereby intensifying the heat transfer process. Liang et al. [9] experimentally evaluated the fluid flow and heat transfer performances of Kagome, body-centered, face-centered, and X-shaped lattices, concluding that X-shaped lattices offer optimal thermal performance. Similarly, Ho et al. [10] embedded aluminum lattice structures into open-slot cold plates, achieving improved local and average heat transfer coefficients. The lattices strengthen the local turbulence intensity and enhance flow mixing, which provides higher thermal performances [11]. However, despite these advancements, conventional lattices are limited by inherent defects such as stress concentration at nodes and low specific surface area [12,13], which hinder their application in spacecraft thermal management. In addition, due to the complex geometry of lattices, the CAD-based design approach is time-consuming for the design of thermal protection systems [14].

Triply periodic minimal surfaces (TPMS) are periodic implicit surfaces with a mean curvature of zero. These surfaces are commonly found in natural structures, such as butterfly wings and beetle skeletons [15]. After millions of years of natural selection, TPMS structures exhibit exceptional properties, including high specific strength, large specific surface area, and high porosity. Based on the connectivity of their internal pores, TPMS lattice structures can be categorized into network-based and sheet-based [16]. Network TPMS lattices contain a single continuous pore region, whereas sheet-based TPMS lattices partition the pore space into two separate and non-intersecting regions, providing a larger specific surface area. The intricate internal channels of TPMS lattice structures enhance heat transfer by increasing the heat transfer area, promoting fluid mixing and turbulence [17]. Furthermore, compared to conventional lattice structures, the smooth and continuous surfaces of TPMS lattices reduce pressure loss, facilitating fluid flow. Noteworthy, the design approach of TPMS lattices is based on mathematical formulae. The surfaces of TPMS lattices are defined by the implicit functions [18]. Therefore, the geometries, such as topology configuration [19], volume fraction [20], and unit cell size [21], are easily modified by changing the parameters of the implicit functions. The mathematically driven design approach makes the TPMS lattices more suitable for the complex thermal protection systems with desirable thermo-fluid properties. In addition, compared to the traditional cooling channels, the TPMS lattices increase the surface area, which is a benefit for thermal management requirements. In summary, the design of novel TPMS-based heatsinks could address the challenges of spacecraft thermal management.

Recently, some researchers have investigated the heat transfer and fluid flow characteristics of TPMS lattices. For example, Renon et al. [22] investigated the thermohydraulic performances of diamond and gyroid TPMS lattices. The simulation results showed that the diamond TPMS lattices had less pressure drop than gyroid ones, which were the optimal choice for heat exchangers. Moradmand et al. [23] found that with an increase in the number of unit cells, the heat transfer coefficient and performance evaluation coefficient of TPMS lattices were improved due to the increase in the surface heat transfer. Lai et al. [24] evaluated the effects of bulk materials on the heat transfer rate of gyroid lattices, and the gyroid heat exchanger made of aluminum increased the heat transfer rate by 6.37% compared to the one made of 316L stainless steel. In addition, researchers have proposed some novel design approaches to further improve the thermo-fluid properties of TPMS lattices, such as functionally graded design [25] and geometric structure control [26].

It should be noted that many studies focused on the investigations of TPMS lattices that possessed a certain topology configuration [26] or a constant volume fraction [27]. A systematic investigation into the fluid flow and heat transfer characteristics of TPMS lattices with different topology configurations and volume fractions is still lacking, and the potential enhancement of thermal performance of TPMS lattices in thermal protection systems remains unclear. Therefore, this study designed three types of TPMS lattices driven by mathematical formulae. The convective heat transfer model for TPMS lattices made of aluminum alloy under single-phase coolant flow was established, which was further validated by experiments. The flow characteristics, heat transfer properties, and overall thermal transfer performance of TPMS lattices were analyzed and compared to traditional RF structures. The TPMS lattices demonstrated excellent heat transfer performances, validating the advantages of TPMS designs in thermal engineering applications.

## 2. Materials and Methods

### 2.1. Lattice Design

**Figure 1:**
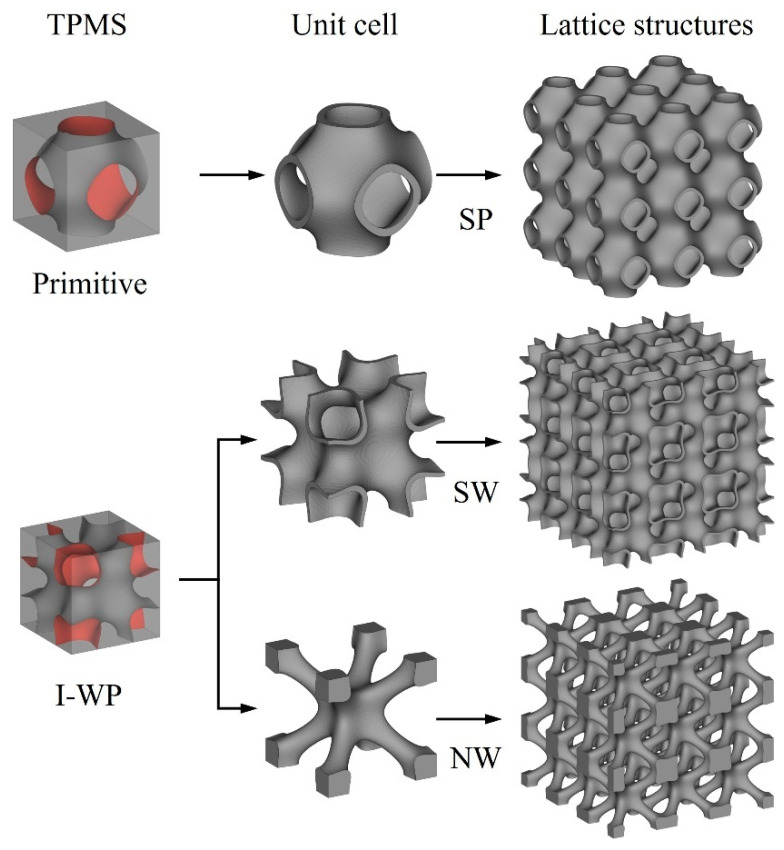
Schematic diagram of TPMS lattices.

TPMSs are periodic surfaces with a mean curvature of zero. These surfaces can be mathematically described using the mathematical function. In this study, the TPMS acts as the boundary between solid and void regions in the design domain. As shown in Figure 1, the TPMS represents a lattice with complex geometries that defines the interface between material and void. Using this method, three types of TPMS lattices were selected for investigation, including sheet primitive (SP), network I-WP (NW), and sheet I-WP (SW), and their formulae are as follows [28]:

(1) $$\varphi_{SP}\left(x,y,z\right)={\left\{\cos\left(ax\right)+\cos\left(ay\right)+\cos\left(az\right)+0.51\left[\cos\left(ax\right)\cos\left(ay\right)+\cos\left(ay\right)\cos\left(az\right)+\cos\left(az\right)\cos\left(ax\right)\right]\right\}}^{2}-t^{2}\leq 0$$

(2) $$\varphi_{NW}\left(x,y,z\right)=\cos\left(2ax\right)+\cos\left(a2y\right)+\cos\left(2az\right)-1.95\left[\cos\left(ax\right)\cos\left(ay\right)+\cos\left(ay\right)\cos\left(az\right)+\cos\left(az\right)\cos\left(ax\right)\right]-t\leq 0$$

(3) $$\varphi_{SW}\left(x,y,z\right)={\left\{\cos\left(2ax\right)+\cos\left(a2y\right)+\cos\left(2az\right)-1.95\left[\cos\left(ax\right)\cos\left(ay\right)+\cos\left(ay\right)\cos\left(az\right)+\cos\left(az\right)\cos\left(ax\right)\right]\right\}}^{2}-t^{2}\leq 0$$

where parameter a controls the unit cell size, and the dimension of unit cells L is determined by [29]

(4) $$L=\frac{2\pi }{a}$$

The volume fraction (VF) of the lattice is computed by integrating the TPMS function:

(5) $$VF=\frac{∭\left(\varphi (x,y,z)\leq 0\right)\cap \left(\left|x\right|\leq \frac{L}{2}\right)\cap \left(\left|y\right|\leq \frac{L}{2}\right)\cap \left(\left|z\right|\leq \frac{L}{2}\right)dxdydz}{L^{3}}$$

The geometric parameters of the TPMS lattices and traditional RF structures are listed in Table 1.

### 2.2. Numerical Method

#### 2.2.1. Mathematical Model

**Figure 2:**
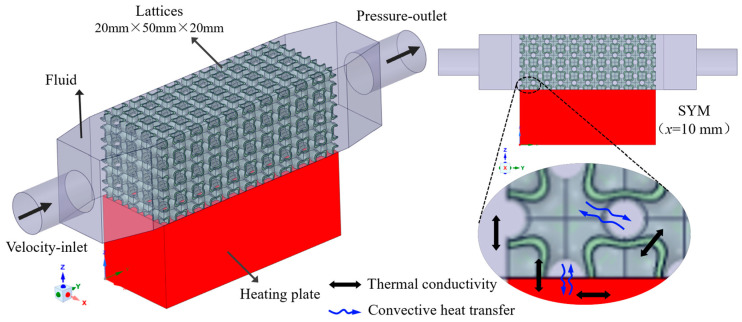
Flow and heat transfer simulation model for TPMS lattices.

To simulate the flow and heat transfer characteristics of TPMS lattices, a computational model was developed in FLUENT, as illustrated in Figure 2. The model consists of a fluid domain, a constant power heat source, and different types of TPMS lattices. A copper plate at the bottom represents a uniform and stable heat source. The size of the copper plate is the same as that used in experiments. To minimize pressure losses caused by unoptimized flow, tapered inlet and outlet regions are added between the fluid domain and the TPMS structures. The coolant (water) flows through the TPMS lattices at a set inlet flow rate, while the copper heating plate uniformly generates heat at a constant power. Heat is transferred from the copper plate to the fluid via conduction and convection, eventually dissipating through the outlet.

The simulation model is governed by the conservation of mass, momentum, and energy. The governing equations have been well documented in previous studies [30,31], and expressed as follows:

Continuity equation:

(6) $$\frac{\partial u_{i}}{\partial x_{i}}=0$$

where ρ is the density of the fluid, $u_{i}$ is the velocity vector components (u, v, and w), and $x_{i}$ is the Cartesian coordinate axis (x, y, and z).

Conservation of momentum equation:

(7) $$\frac{\partial }{\partial x_{j}}\left(\rho u_{i}u_{j}\right)=-\frac{\partial p}{\partial x_{i}}+\frac{\partial }{\partial x_{j}}\left[\left(\mu +\mu_{t}\right)\left(\frac{\partial u_{i}}{\partial x_{j}}+\frac{\partial u_{j}}{\partial x_{i}}\right)\right]$$

where p is the pressure, μ is the dynamic viscosity of the fluid, and $\mu_{t}$ is the turbulent viscosity.

Energy equation:

(8) $$\left\{\begin{array}{c} \frac{\partial }{\partial x_{j}}\left(\rho u_{i}T\right)=\frac{\partial }{\partial x_{i}}\left[\left(\frac{k}{C}+\frac{\mu_{t}}{Pr}\right)\frac{\partial T}{\partial x_{i}}\right]\;(\mathrm{For}\;\mathrm{fluid}\;\mathrm{domain})\\ \frac{\partial }{\partial x_{j}}\left(\frac{\partial T_{S}}{\partial x_{j}}\right)=0\;(\mathrm{For}\;\mathrm{solid}\;\mathrm{domain}) \end{array}\right.\;$$

where T is the fluid temperature, k is the thermal conductivity of the fluid, C is the specific heat of the fluid, Pr is the turbulent Prandtl number, and $T_{S}$ is the solid temperature.

Due to the complex internal geometry of TPMS lattices, vortex formation and turbulence occurred during fluid flow. The RNG k − ε turbulence model was selected for the simulation to capture rotational and swirling flows [32]. The commercial computational fluid dynamics software, ANASYS FLUENT 2020 R1, was used to numerically solve the governing equations. The material properties used in the simulation are listed in Table 2, with water as the coolant, AlSi10Mg for the TPMS lattices, and cast copper for the heating plate. Boundary conditions were set as follows: the inlet was defined as a velocity-inlet with an initial coolant temperature of 293 K, while the outlet was set as a pressure-outlet, as summarized in Table 3. The inlet flow rates of 0.05–0.40 L/min were investigated. The copper plate generated the heat of 100 W with a convection heat transfer coefficient of 8 W·m⁻^2^·K⁻^1^ at the external surfaces.

#### 2.2.2. Mesh Independence Study

**Figure 3:**
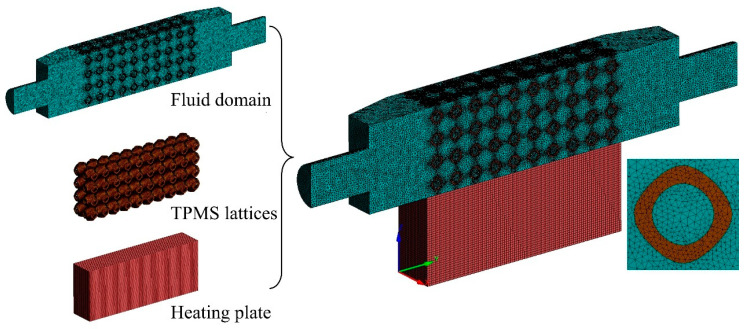
Meshes of the computational domain.

**Figure 4:**
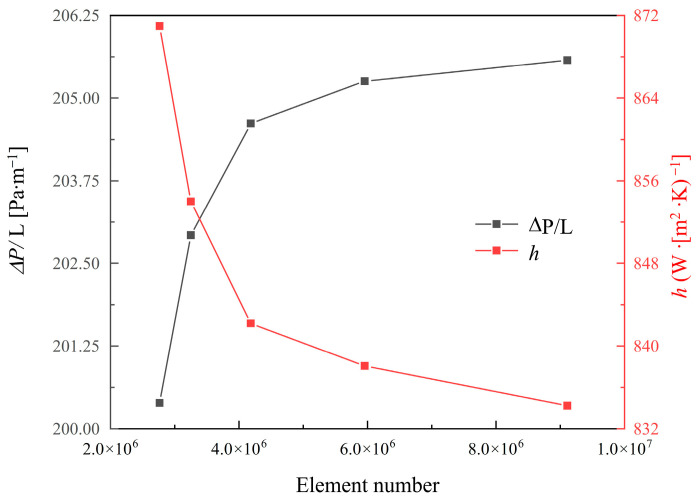
Mesh convergence results for pressure gradient (ΔP/L) and heat transfer coefficient (h).

The model of TPMS lattices was generated by a Python 3.0 code and meshed using SpaceClaim. The computational domain for the TPMS lattices was meshed using unstructured tetrahedral elements for the solid and fluid domains, while hexahedral elements were used for the heating plate. Figure 3 illustrates the meshed computational domain. To ensure accuracy, a mesh convergence analysis was performed, and the pressure gradient and convective heat transfer coefficient were used as evaluation criteria. As shown in Figure 4, when the total number of mesh reached approximately 6,000,000, the pressure gradient error dropped below 0.5%, and the convective heat transfer coefficient error was within 0.95%. Therefore, 6,000,000 elements were selected for the simulation models. All simulations were performed on an Intel Core i9-13900K CPU (Intel, Santa Clara, CA, USA) with 24 cores and 32 GB RAM, and the computer time was approximately 1 h for each model.

### 2.3. Mathematical Analysis

To assess the flow characteristics, heat transfer properties, and overall thermal performance of the TPMS lattices, several indexes were introduced as follows:

(1) Flow characteristics

The pumping power required to transport the coolant can be expressed as:

(9) $$W=\Delta P\cdot Q_{v}$$

where $Q_{v}$ is the fluid volumetric flow rate, and ΔP is the pressure loss between the inlet $P_{in}$ and outlet $P_{out}$ of the heat sink, defined as:

(10) $$\Delta P=P_{in}-P_{out}$$

Permeability is a key indicator used to assess the flow characteristics of lattices. According to Darcy’s law, when the Reynolds number (Re) is less than 10, the ΔP and flow velocity (v) of the porous structures exhibit a linear relationship, expressed as [33]:

(11) $$\frac{\Delta P}{L}=\frac{\mu }{K}v$$

where ΔP/L is the pressure gradient along the flow direction, μ is the dynamic viscosity of the fluid, and K is the permeability of the lattices. In this study, the v is the mean fluid velocity at the inlet surface. When the Re is larger than 10, the ΔP and v follow a nonlinear relationship, described by the Darcy–Forchheimer law [34]:

(12) $$\frac{\Delta P}{L}=\frac{\mu }{K}v+\rho Cv^{2}$$

where ρ is the fluid density, and C is the resistance coefficient. The Fanning friction factor (f) is a dimensionless parameter used to characterize the resistance caused by fluid viscosity and vortices during heat dissipation, which is defined as [35]:

(13) $$f=\frac{2\Delta PD}{L\rho v^{2}}$$

where L is the length of the porous structures the fluid flows through, and D is the equivalent diameter. Considering the complex internal structures of TPMS lattices, the D is defined as:

(14) $$D=\frac{2WH}{W+H}$$

where W and H are the width and length of the TPMS lattices at the inlet. Then, *Re* is calculated by:

(15) $$Re=\frac{\rho vD}{\mu }$$

(2) Heat transfer characteristics

The convective heat transfer coefficient (h) is used to evaluate the heat transfer ability between the fluid and the solid wall. Since the fluid temperature on the fluid side is difficult to measure experimentally, the temperature at the inlet is used to calculate h, defined as:

(16) $$h=\frac{q}{T_{w}-T_{f}}$$

where q is the transferred heat flux, and $T_{w}$ and $T_{f}$ is the temperature difference between the wall and the fluid, respectively. The Nusselt number (Nu) is a dimensionless parameter that describes the intensity of heat transfer from the fluid to the solid surface. Nu can characterize the enhancement or suppression of heat transfer due to fluid flow, expressed as:

(17) $$Nu=\frac{hD}{\lambda }$$

where λ is the thermal conductivity of the fluid. The local Nu along the flow direction at the solid–liquid interface can be expressed as:

(18) $${Nu}_{Y}=\frac{h_{Y}D}{\lambda }=\frac{\partial \left[\left(T_{w}-T)/(T_{f}-T\right)\right]}{\partial (Y/D)}$$

where Y represents the coordinate along the flow direction of the coolant. The dimensionless heat transfer factor (j) is used to measure the heat transfer performance of the system and is defined as:

(19) $$j=\frac{Nu}{Re\cdot {Pr}^{1/3}}$$

where Pr is the Prandtl number and calculated as:

(20) $$Pr=\frac{C\mu }{\lambda }$$

(3) Overall thermal transfer performance

To evaluate the overall thermal performance of TPMS lattices, the dimensionless f and j are combined into an overall performance factor (α). This factor is based on the characteristic that the pressure gradient increases quadratically with flow velocity. It can be used to assess whether the improvement in heat dissipation ability outweighs the increase in flow resistance under constant pressure drop. The definition of α is:

(21) $$\alpha =\frac{f}{j^{1/3}}$$

### 2.4. Sample Fabrication

**Figure 5:**
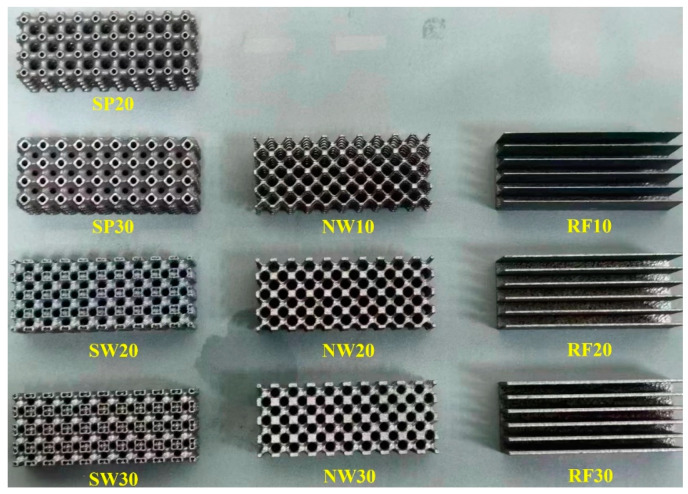
Fabricated TPMS lattice and rectangular fin samples using selective laser melting.

The TPMS lattices were fabricated using a commercial laser powder bed fusion (LPBF) machine (XDM 250) (XDM, Suzhou, China) with AlSi10Mg powder. The process parameters included a powder layer thickness of 30 µm, a laser power of 375 W, and a scanning speed of 1600 mm/s. Argon gas was used for inert protection, maintaining an oxygen content of less than 0.1%. Figure 5 shows the fabricated TPMS and RF samples.

### 2.5. Experiments Details

**Figure 6:**
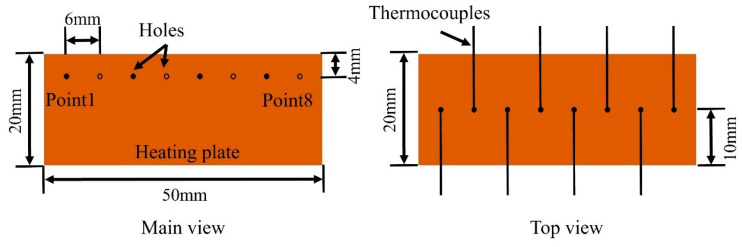
Thermocouple arrangement on the heating plate for temperature measurements.

The experiments measured the steady-state temperature of the heating plate. Thermocouples measured the temperature of the heating plate at specific locations, as demonstrated in Figure 6. During the testing, coolant water at an inlet temperature of 20 °C was circulated through the samples. Once the temperature was stable, the copper heating plate was activated, and the temperature distribution was monitored.

## 3. Results and Discussion

### 3.1. Validation of Simulations

**Figure 7:**
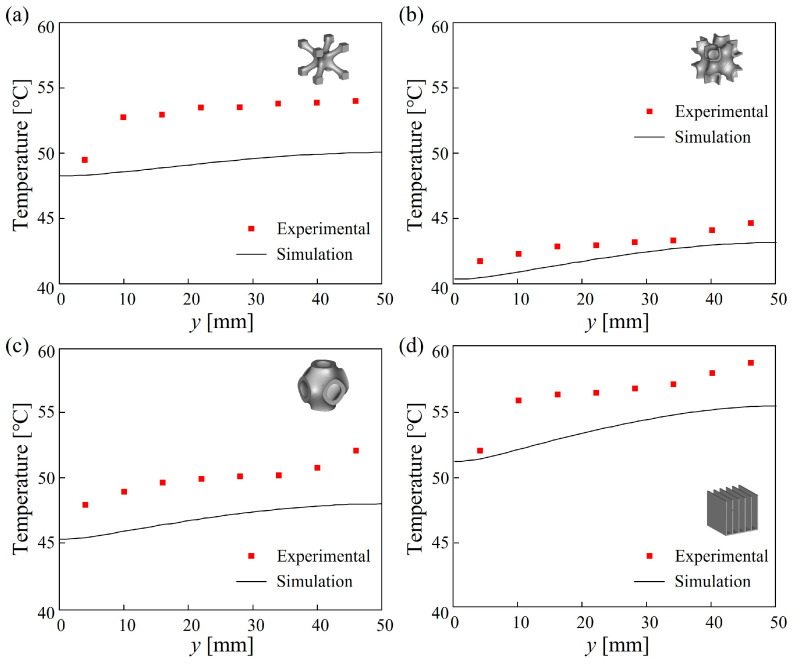
Temperatures on the heating plate under steady-state heat transfer conditions: (**a**) NW20 (**b**) SW20 (**c**) SP20 (**d**) RF20.

Figure 7 shows the experimental and simulated temperatures at various measurement points on the heating plate for TPMS lattices and the traditional RF structure under steady-state heat transfer conditions. As cooling water flowed through the TPMS lattices, it exchanged heat with the solid framework and the baseplate, leading to a gradual increase in temperature. Therefore, the outlet regions exhibited the highest temperature. Compared to the simulation results, the measured temperatures at each point are slightly higher than the simulated values. The deviation is because the simulation model neglected the thermal resistance between the sample and the heating plate. In the experiments, due to surface roughness, the sample and the heating plate could not achieve perfect contact, resulting in contact thermal resistance at the solid–liquid interface. This resistance will weaken the heat dissipation efficiency of the samples. Overall, the simulation model successfully predicted the temperature rise characteristics along the flow direction, with a maximum error of less than 8.5%. As shown in Figure 7, the temperatures of the TPMS samples were all lower than those for the RF sample, and the order of the highest temperatures is RF > NW > SP > SW. This trend was also successfully predicted by simulations.

### 3.2. Fluid Flow Characteristics

#### 3.2.1. Fluid Flow Mechanisms

**Figure 8:**
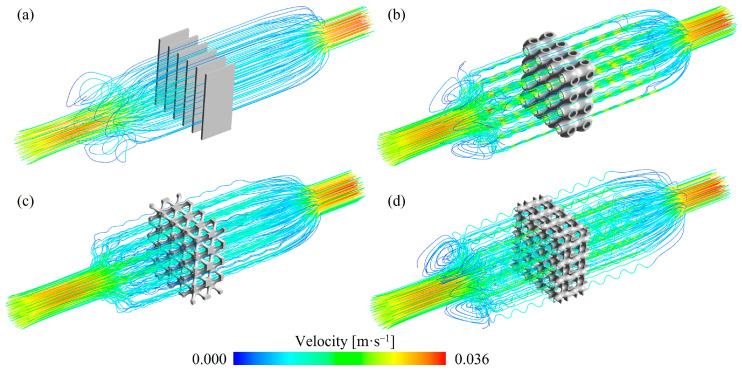
Streamlines within TPMS lattices and RF structures with 0.2 volume fraction: (**a**) RF20 (**b**) SP20 (**c**) NW20 (**d**) SW20.

Figure 8 illustrates the streamlines within TPMS lattices and RF structures with a VF of 0.2. Compared to the RF structures, the fluid streamlines in the TPMS lattices were more tortuous. Among the TPMS lattices, the streamlines in the SW20 and SP20 lattices were more complex than those in the NW20 lattices. This difference arises from the correlation between the complexity of fluid pathways and the structural intricacy. For SW20 and SP20 lattices, the fluid was divided into two independent channels, which increased the tortuosity of the streamlines.

**Figure 9:**
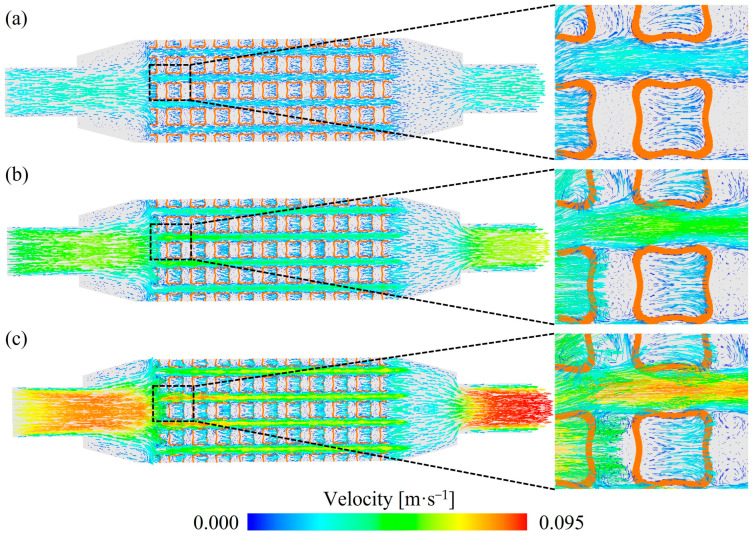
Velocity fields of the SW20 lattices under different flow rates at *z* = 30 mm cross-section: (**a**) 0.1 L/min (**b**) 0.2 L/min (**c**) 0.3 L/min.

To further analyze the fluid flow mechanisms of TPMS lattices, the velocity fields of the SW20 lattices under different flow rates at *z* = 30 mm cross-section are shown in Figure 9. Due to the obstruction caused by the aluminum skeletons of the SW20 lattices, significant vortices were generated near the inlet region. A similar phenomenon was also found in the gyroid lattice structures [36]. These vortices helped distribute the fluid velocity more evenly as it entered the internal pores. Within the porous regions, the velocity distribution was relatively uniform and lower than in the inlet and outlet regions. Near the lattice surfaces, the flow velocity approached 0 m/s due to the viscous forces imposed by the no-slip boundary condition, which introduced significant drag as the fluid interacted with the solid surface. Along the flow direction, the cross-sectional area of the SW20 lattices periodically varied. When the cross-section reduced, the fluid velocity increased. These periodic changes in the cross-section induced secondary flow and intense disturbances, influencing the velocity magnitude and direction of the fluid. Local vortices were continuously formed as the fluid separated and re-converged. As demonstrated in Figure 9, the fluid velocity increased with the increase of the inlet flow rate, and the regions of flow vortices were expanded accordingly.

#### 3.2.2. Fluid Flow Resistance

**Figure 10:**
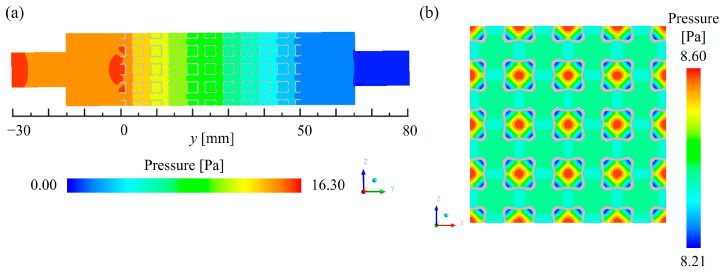
Pressure distribution of the SW20 lattices: (**a**) *x* = 10 mm (**b**) *y* = 25 mm.

Figure 10 shows the pressure distribution of the SW20 lattices at the *x* = 10 mm and *y* = 25 mm cross-sections. As demonstrated in Figure 10a, the pressure in the inlet region of the lattices (*y* = −30~0 mm) was higher than in the outlet region (*y* = 50~80 mm) due to the direct impact of the incoming fluid. The pressure gradually decreased along the flow direction, with most of the pressure loss concentrated in the lattices. In contrast, the pressure loss in the inlet and outlet regions was uniform, indicating that the TPMS lattices were the primary cause of pressure reduction. The pressure drop in the lattices is mainly attributed to two factors. First, local pressure loss caused by fluid impact on the solid regions of the lattices. Second, frictional pressure loss induced by the interaction between the fluid and the lattice surface. As shown in Figure 10b, the pressure was relatively uniform within the porous regions, while pressure gradients primarily concentrated near the lattice surfaces. Moreover, the regions closer to the solid surfaces exhibited a lower pressure, while the pressure in the center of the pores was relatively higher. This confirms that the TPMS lattices were the primary factor influencing the pressure variations.

**Figure 11:**
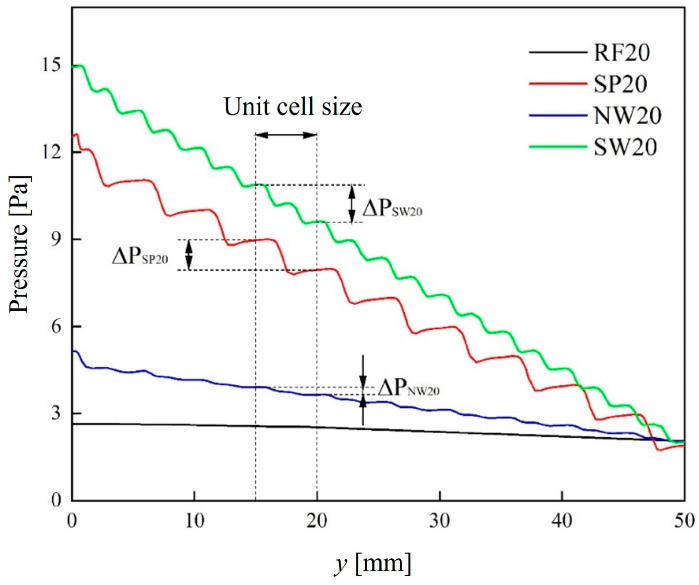
Pressure curves along the flow direction for TPMS lattices and the RF structure with 0.2 volume fraction.

Figure 11 presents the pressure curves along the flow direction for TPMS lattices and the RF structure with 0.2 volume fraction. For the RF structures, the pressure decreased linearly and gradually along the flow direction. In contrast, the pressure of TPMS lattices exhibited a stepwise decline. Once the flow stabilized, the pressure loss of the TPMS lattices also stabilized. The pressure losses for the SP, NW, and SW lattices were 1.01 Pa, 0.26 Pa, and 1.27 Pa, respectively.

**Figure 12:**
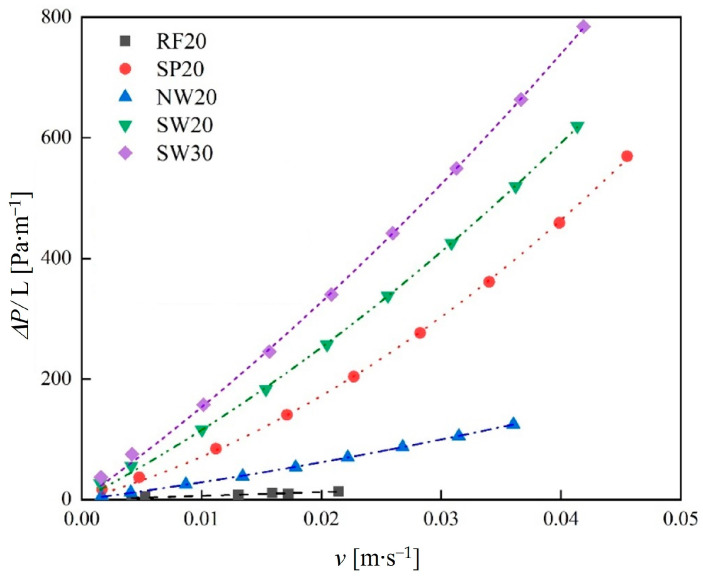
Relationship between pressure gradient and flow velocity for TPMS lattices and the RF structures. (Inlet flow rates of 0.05–0.40 L/min).

Figure 12 illustrates the relationship between pressure gradient and flow velocity for TPMS lattices and the RF structures. The results show that the pressure gradient of TPMS lattices was higher than that of the RF structures at the same flow velocity. This is due to the higher number of internal pores in TPMS lattices and their tortuous flow paths, which increased the fluid flow length and the solid–fluid contact area. As a result, fluid flowing through TPMS lattices experienced the higher resistance. Moreover, at the same flow velocity, increasing the volume fraction led to the higher pressure gradient. This is because reducing the pore size within TPMS lattices hindered fluid flow, and the larger solid regions further obstructed the fluid.

As shown in Figure 12, the pressure gradient increased with the flow velocity for TPMS lattices. At low flow velocities, the pressure drop was primarily influenced by viscous friction, which is related to the solid–fluid contact area. As the flow velocity increased, the relationship between the pressure gradient and flow velocity deviated from linearity, and the pressure drop was mainly affected by the inertial forces. Therefore, the Darcy–Forchheimer law was used to fit the relationship between the pressure gradient and flow velocity of TPMS lattices. The R^2^ for all fitted equations exceeded 0.99, indicating a high accuracy. The results show that the pressure gradient in TPMS lattices increased quadratically with flow velocity.

According to Equation (12), the permeability (K) and resistance coefficient (C) of the TPMS lattices were calculated, as shown in Table 4. The results indicate that for a constant volume fraction, the NW lattices exhibited the highest K and the lowest C, suggesting that fluid flows more easily through the NW lattices. However, as the volume fraction increased, the K decreased and the C increased. These findings further confirm that at low flow velocities, the pressure drop is primarily dominated by viscous friction, while at higher flow velocities, inertial forces play a more significant role.

**Figure 13:**
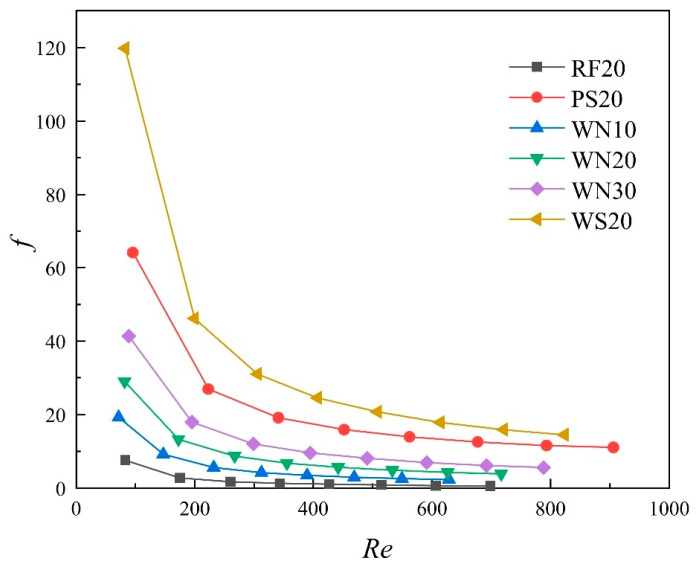
Relationship between the Fanning friction factor (f) and the Reynolds number (Re) of TPMS lattices. (Inlet flow rates of 0.05–0.40 L/min).

Figure 13 illustrates the relationship between the Fanning friction factor (f) and the Reynolds number (Re) for TPMS lattices under different inlet flow rates. It can be observed that f continuously decreased as Re increased. Particularly for the low Re, f significantly dropped as Re increased. However, when Re increased to approximately 400, the degree of decrease in f became more gradual. This indicates that as the flow rate increased beyond a certain threshold, the effects of viscous forces and vortex-induced resistance decreased, and the pressure drop became predominantly influenced by inertial forces. Additionally, f increased with the increase in the volume fraction of the lattices. This is mainly due to the increased surface area for the lattices with large volume fractions, leading to the higher frictional resistance.

### 3.3. Heat Transfer Characteristics

#### 3.3.1. Temperature Distribution

**Figure 14:**
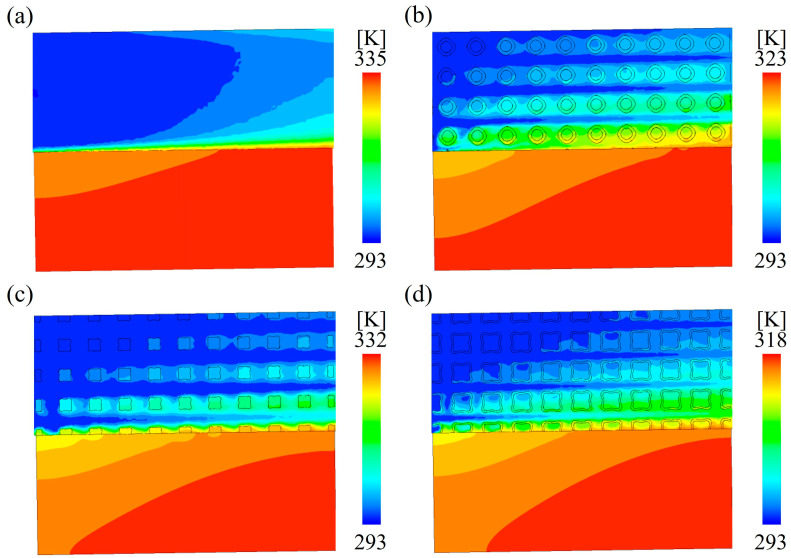
Temperature distribution of samples at the *x* = 10 mm section: (**a**) RF20 (**b**) SP20 (**c**) NW20 (**d**) SW20.

Figure 14 shows the temperature distribution of the TPMS lattices and traditional RF structures at the *x* = 10 mm cross-section under the same flow rate. Because the solid regions of the TPMS lattices and FS structures were directly in contact with the top surface of the copper plate, the heat was transferred via conduction. As the low-temperature coolant flowed over the high-temperature solid regions and the surface of the heating plate, the heat was transferred to the fluid through convection, causing the temperature of the fluid to gradually rise. As a result, along the direction of fluid flow, the convective heat transfer effect gradually diminished, leading to a parabolic increase in the temperature of the heating plate along the flow direction. Ultimately, heat accumulation occurred at the end of the heating plate, developing into a local high-temperature concentration. Compared to the traditional RF structures, the TPMS lattices exhibited a more uniform temperature distribution and a lower maximum temperature, indicating that the heat transfer performance of the TPMS lattices was superior to that of the traditional RF structures. The superior heat transfer performance of the TPMS lattices is because the fluid tended to form vortices as it flowed through the complex internal structures, which increased turbulence intensity and enhanced the convective heat transfer between the TPMS lattices and the cooling fluid. Similarly, the previous studies found that the geometry of lattice structures significantly influenced the fluid mixing effect on the heating surface and further changed the turbulent effects and influenced the local overheating [37]. Additionally, the convective heat transfer area of the TPMS lattices was larger than that of the traditional RF structures, further improving the convective heat transfer performance. Among the three types of TPMS lattices, SW lattices showed the best heat dissipation performance due to their complex internal structures and largest specific surface area.

**Figure 15:**
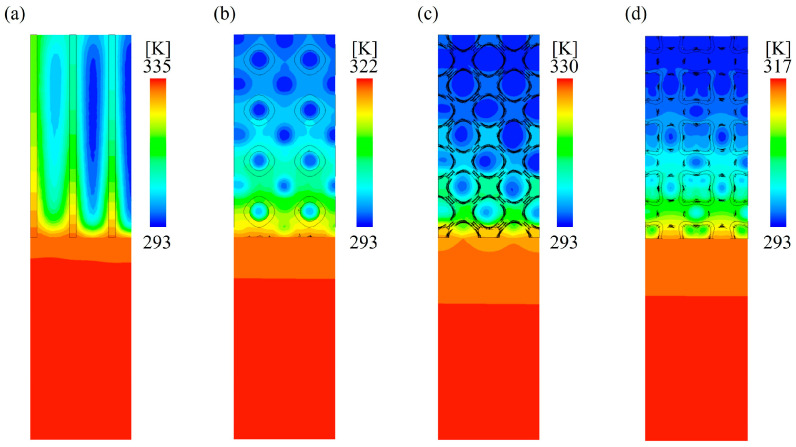
Temperature distribution of samples at the *y* = 25 mm cross-section: (**a**) RF20 (**b**) SP20 (**c**) NW20 (**d**) SW20.

Figure 15 shows the temperature distribution of TPMS lattices and the RF structures at the *y* = 25 mm cross-section under the same flow rate. The results indicate that temperature gradients mainly appeared across the solid lattices and the fluid domain. Within the fluid domain, the temperature in the pore regions was close to the inlet fluid temperature, while the temperature near the solid regions of the lattices was significantly higher.

**Figure 16:**
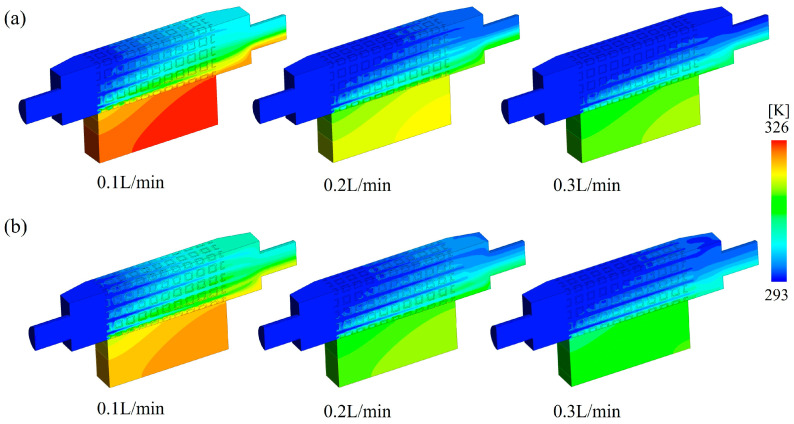
Temperature distribution of SW lattices with volume fractions of (**a**) 0.2 and (**b**) 0.3 under different inlet flow rates.

Figure 16 compares the temperature distribution of SW lattices with volume fractions of 0.2 and 0.3 under different inlet flow rates. The results reveal that increasing the inlet flow rate enhanced the heat removal capacity of the lattices. At the same flow rate, the SW30 lattices exhibited superior heat dissipation compared to the SW20 ones. This is because the SW30 lattices had a larger surface area for convective heat transfer and a greater contact area with the heating plate. Moreover, the smaller pore size within the SW30 lattices increased the local flow velocity, thereby intensifying both conductive and convective heat transfer. These factors enable the fluid to remove more heat within the same timeframe for SW30 lattices, effectively reducing the temperature of the baseplate.

#### 3.3.2. Convective Heat Transfer Properties

**Figure 17:**
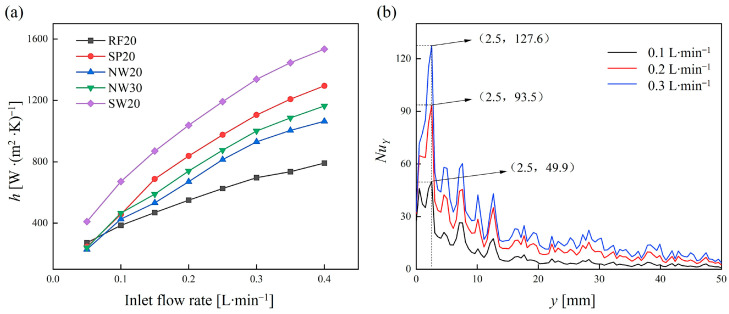
(**a**) Relationship between the convective heat transfer coefficient (h) and the inlet flow rate. (**b**) Variation in the local Nusselt number (Nu) along the flow direction for the SW20 lattices under different flow rates.

Figure 17a shows the relationship between the convective heat transfer coefficient (h) and the inlet flow rate for TPMS lattices and RF structures. Generally, the convective heat transfer coefficient increased with the increase in the inlet flow rate, but the rate of increase gradually slowed down. This indicates that the enhancement of convective heat transfer became weaker as the inlet flow rate increased. Compared to traditional RF structures, the TPMS lattices had a higher convective heat transfer coefficient, which can be increased by up to approximately 96.62%. This is due to the complex internal structures of the TPMS lattices, which enhanced the disturbance of the fluid flow. Furthermore, the order of the convective heat transfer coefficients of the TPMS lattices was SW > SP > NW, which is consistent with the complexity of their internal structures and surface areas. For the TPMS lattices with the same topology configuration, increasing the volume fraction led to an increase in heat transfer performance. This improvement can be attributed to two main factors: First, the increased contact area between the lattice and the heating plate enhanced thermal conduction. Second, the reduced pore size raised the fluid velocity within the channels under the same inlet flow rate, allowing more heat to be carried away.

Figure 17b shows the variation in the local Nusselt number (*Nu*) along the flow direction for the SW20 lattices under different flow rates. The results demonstrate that Nu increased with the inlet flow rate, indicating that the intensity of convective heat transfer improved as the flow rate increased. Along the flow direction, the Nu rapidly increased to an initial peak in the inlet region, followed by a large drop with fluctuations. This phenomenon can be attributed to the twisted flow channels within the TPMS lattice unit cells, which promote fluid mixing and induce localized recirculation. These effects intermittently disrupted the growth of the boundary layer, leading to intensified convective heat transfer at certain locations.

### 3.4. Overall Thermal Transfer Performance

**Figure 18:**
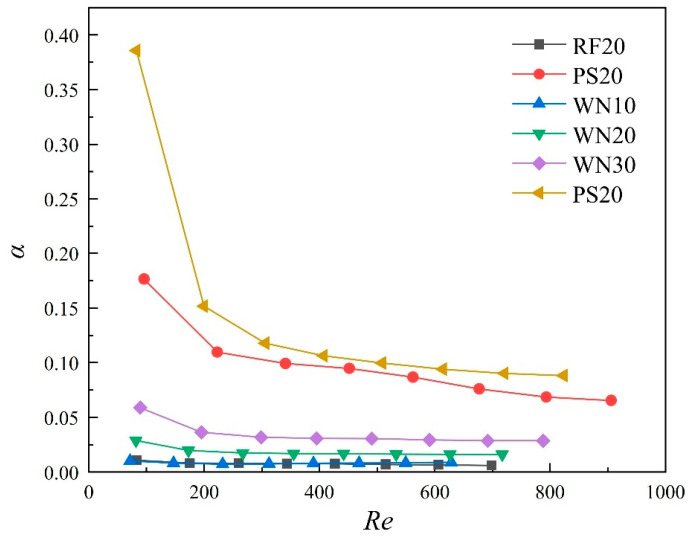
Overall heat transfer performance index (α) of TPMS lattices and RF structures. (Inlet flow rates of 0.05–0.40 L/min).

The overall heat transfer performance index (α) of the TPMS lattices and traditional RF structures was calculated using Equation (21), and the results are shown in Figure 18. It can be seen that as the Re increased, the α of all samples gradually decreased. The decreasing trend of the α indicates that the power consumption increased continuously during heat transfer enhancement. At low Re, the heat transfer capability of the samples was less enhanced than the increase in structural resistance. As the Re increased, the heat transfer enhancement became lower, and the inertial forces in the flow resistance became more significant. Additionally, the viscous forces, which are mainly affected by the structural features, become smaller. Therefore, the α decreased slowly and entered a stable trend. For the TPMS lattices with the same topology configuration, increasing the volume fraction helped to improve the α. Overall, the α of the TPMS lattices was significantly higher than that of the traditional RF structures, with SP > SW > NW, confirming the potential applications of TPMS lattices in thermal protection systems.

## 4. Conclusions

Inspired by the minimal surfaces in nature, this study designed three types of TPMS lattices for heat dissipation using mathematical formulae. The convective heat transfer model for TPMS lattices made of aluminum alloy under single-phase coolant flow was established, which was further validated by experiments. The fluid flow and heat transfer characteristics of TPMS lattices were simulated and compared to traditional RF structures. Based on the simulation results, the flow characteristics, heat transfer properties, and overall thermal performances of TPMS lattices were investigated. The main conclusions are as follows:

(1) Compared to traditional RF structures, TPMS lattices had larger convective heat transfer areas and more complex internal configurations, which enhance the frictional resistance of the fluid. These features generated distinct flow vortices and high flow resistance for TPMS lattices.

(2) The pressure gradient of TPMS lattices followed a quadratic relationship with flow velocity, satisfying the Darcy–Forchheimer law. Among the three TPMS lattices, the NW lattices had the highest permeability and the lowest resistance coefficient. As the volume fraction increased, the permeability decreased and the resistance coefficient increased.

(3) Compared to traditional RF structures, TPMS lattices exhibited a more uniform temperature distribution and a higher convective heat transfer coefficient under the same flow conditions. The convective heat transfer coefficient can be further improved by increasing the inlet flow rate and the volume fraction.

(4) The SP and NW lattices exhibited the highest and lowest overall thermal performance, but the overall thermal performance of all TPMS lattices was higher than that of traditional RF structures, which validates the potential applications of TPMS lattices in thermal protection systems.

## Figures and Tables

**Table 1:** Geometric parameters of TPMS lattice and traditional RF structures.

Sample	Type	*VF*	Surface Area [mm^2^]	Dimensions [mm × mm × mm]
SP 20	Sheet primitive (SP)	0.2	18,566	20 × 50 × 20
SP 30		0.3	18,367	
SW 20	Sheet I-WP (SW)	0.2	27,969	
SW 30		0.2	27,228	
NW 10	Network I-WP (NW)	0.1	8665	
NW 20		0.2	11,716	
NW 30		0.3	13,321	
RF10	Rectangular fins (RF)	0.1	6180	
RF20		0.2	6360	
RF30		0.3	6540	

**Table 2:** Material properties used in the simulation.

Material	Specific Heat [J·(kg·K)^−1^]	Thermal Conductivity [W·(m·K)^−1^]	Density [kg·m^−3^]	Dynamic Viscosity [Pa·s]
Water	4180	0.599	998.2	1.003 × 10^−3^
Cast copper	381	387.6	8978	
AlSi10Mg	895	151.468	2670	

**Table 3:** Thermal and fluid flow boundary conditions.

**Boundary**	Velocity	Pressure	Temperature
Inlet	*u* = *w* = 0 $v=\frac{Re\mu }{P_{f}D}$	-	$T_{f}=293K$
Outer	-	P=0	-

**Table 4:** Permeability (K) and resistance coefficient (C) of TPMS lattices.

Coefficient	SW30	SW20	SP20	NW20
K [m^2^]	7.23 × 10^−8^	9.31 × 10^−8^	1.72 × 10^−7^	3.72 × 10^−7^
C [m^−1^]	117.15	99.14	143.62	24.21

## Data Availability

The original contributions presented in this study are included in the article. Further inquiries can be directed to the corresponding authors.
